# Evaluation of bevelled needle tip deformation with Dental Inferior Alveolar Nerve blocks

**DOI:** 10.1111/aej.12361

**Published:** 2019-07-03

**Authors:** Tony Skapetis, Phuong Diem Doan‐Tran, Nibir M. Hossain

**Affiliations:** ^1^ Faculty of Medicine & Health Sydney Dental School Clin A/Prof University of Sydney Westmead Centre for Oral Health, WSLHD Westmead NSW Australia; ^2^ Faculty of Medicine & Health Sydney Dental School University of Sydney Sydney NSW Australia

**Keywords:** barbing, dental anaesthesia, dental blocks, distortion, inferior alveolar nerve block, needle tip deformation

## Abstract

The study aimed to investigate whether any correlation existed between bevel orientation and needle tip deformation following the administration of a standard inferior alveolar nerve block (IANB) technique during patient treatment. Ninety‐three needles of a single brand were collected from a group of eleven similarly trained Australian dentists’ following either single or dual insertion and bone contact. Specimens were examined under scanning electron microscopy at 500x, and both the direction of deformation (either towards or away from lumen) and the extent of deformation were calculated using image processing software. Results showed no correlation between bevel orientation and either the direction (*P *= 0.8787) or degree (*P* = 0.0752) of deformation. Significance was demonstrated, regardless of bevel orientation, following multiple needle use with respect to extent of needle tip deformation (*P* < 0.0001). A clinical recommendation can be made that the dental needle should be routinely replaced when subsequent injections are required during the delivery of a typical IANB.

## Introduction

Local anaesthesia is an integral part of most operative dental procedures due to its effectiveness in the prevention and management of perioperative and postoperative pain 1, and one of the most commonly used techniques in dentistry is the inferior alveolar nerve block (IANB). The IANB is the most frequently used technique in endodontics for anaesthetising mandibular molar teeth 2 and is associated with a broad spectrum of reported failure rates from low teens to as high as 81% 2. These failures have been attributed to many factors including anatomic variability, treatment type, pathological and psychological causes 3. As a result of anaesthetic inadequacy or failure, subsequent IANB injections are often given without replacement of the needle 3, 4, and following unpublished investigation by the authors, this practice is widely and consistently propagated by the majority of the 9 universities teaching dentistry in Australia.

Postoperative complications such as trismus and paraesthesia have been reported as a result of IANB injections with some suggested aetiologies being direct needle trauma to the nerve, intraneural haematoma formation and neurotoxicity of the local anaesthetic agent itself 5. Other authors have suggested that the tearing of tissues caused by needle tip deformation (also referred to as distortion, deviation and barbing) after contacting bone during IANBs may be a significant contributory factor 6. Additionally, the degree of needle tip deformation may be influenced by such factors as clinician variability, force differential, bevel characteristics and manufacturing differences (7). Furthermore, there appears to be no consistent agreement as to the preferred orientation of the bevel during standard IANB delivery 8. Some authors suggest that it is best to orientate the bevel away from the mandibular ramus to help minimise needle deflection and improve injection accuracy 2 while other suggesting that barbing of the needle tip is of a more favourable nature 9 or less extensive 4 when the bevel is oriented towards the ramus and yet others report no preference, claiming success of IANBs was unaffected by bevel orientation 6.

The purpose of this cross‐sectional clinical study was to identify whether any correlation exists between bevel orientation following single and multiple (twice) needle use during IANB delivery in terms of direction (towards or away from lumen) and extent of needle tip deformation. Secondarily, these results may be used to guide needle use during dental IANB procedures.

## Materials and methods

This study was fully approved by the Local Health District Human Research and Ethics Committee.

This cross‐sectional study collected needle samples from a group of clinicians following IANB delivered during general patient care within a large public teaching hospital. A total of 11 fully registered dentists were voluntarily inducted into the study with eligibility restricted to Australian graduates who completed their studies within the last 5 years. This criterion helped ensure a level of homogeneity amongst study participants, thereby reducing some confounders with respect to IANB technique. Clinicians were aware of the study aims and performed multiple needle insertions dependent on anaesthesia quality. Bevel orientation was not dictated by the study protocol but was to be noted by each participating clinician.

All needles collected were identical in brand, gauge and length (27 gauge, 38 mm Morita disposable dental needles). Each clinician was asked to administer ten IAN block injections – half with the bevel facing towards the ramus of the mandible and half directed away. Needles were further subcategorised according to single or multiple use (twice only). Clinicians were asked to pay special attention to the handling of needles in order to avoid distortion of the needle tip when recapping the needles for collection. To increase the validity of our sample data, all clinicians were provided with a ‘frequently asked questions’ document, which emphasised attention towards bevel orientation and careful sample collection.

Four jars each containing 180 mL of 70% ethanol were used to house the used needles and were marked as follows:

1. Bevel towards bone with only one insertion
2. Bevel towards bone with two insertions
3. Bevel away from bone with one insertion
4. Bevel away from bone with two insertions

A total of 103 needles were evaluated in this study, inclusive of 10 unused controls. Needle specimens were carefully mounted with the aid of a light microscope onto electron microscope stubs, ensuring the bevel was oriented to the left‐hand side so that the needle tip could be observed laterally. Scanning electron microscopy (SEM) was used to take images of the samples at 500x magnification and needle tip deformation calculated using the microscopy image processing program, Image J.

**Figure 1:**
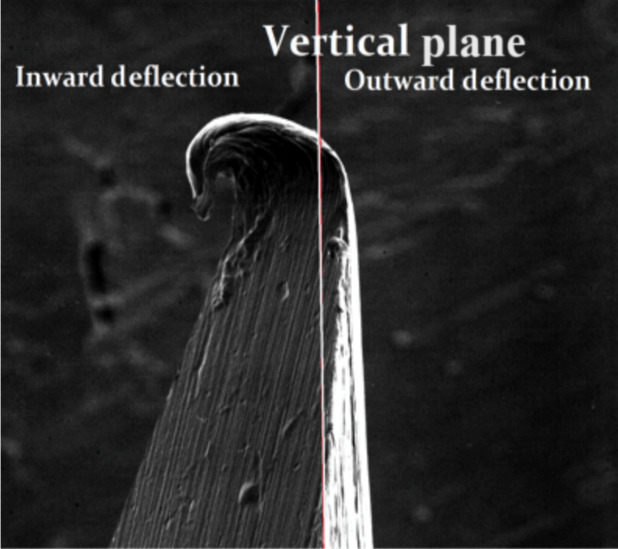
SEM image of needle tip deformation relative to long axis (500x‐).

The direction of needle tip deformation was classified as either inward (towards the lumen of the needle) or outward (away from the lumen) facing, relative to the long axis (see Fig. 1). The extent of needle tip deformation was also measured in terms of angle of deviation, relative to the long axis, from the initial point along the bevel where the deformation began. Negative angulation values were assigned to outward facing and positive values to inward facing deformations, respectively, and data were analysed using GraphPad Prism 7 for Mac.

The variables of bevel orientation and single/dual insertions with their effect on direction and extent of deviation were compared using unpaired *t* tests, and a two‐way anova was used to investigate the effect of bevel orientation and direction of deflection (inward/outward) on the degree of needle tip distortion measured in degree angles.

The analysis of the extent of distortion was completed in two ways, firstly with raw data and degrees measured to two decimal places (raw angle) and secondly using grouped data, where values falling between a set range of angles were clustered together (clustered angle). A *P* value threshold of 0.05 was selected for significance.

## Results

**Figure 2:**
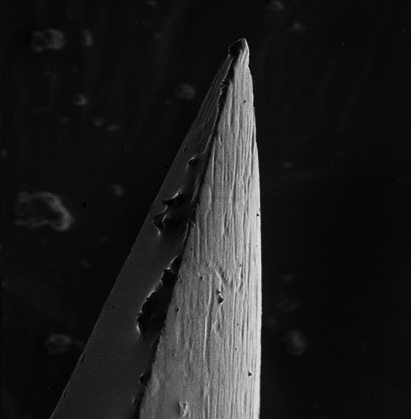
Control sample showing undamaged bevel tip (500x).

**Figure 3:**
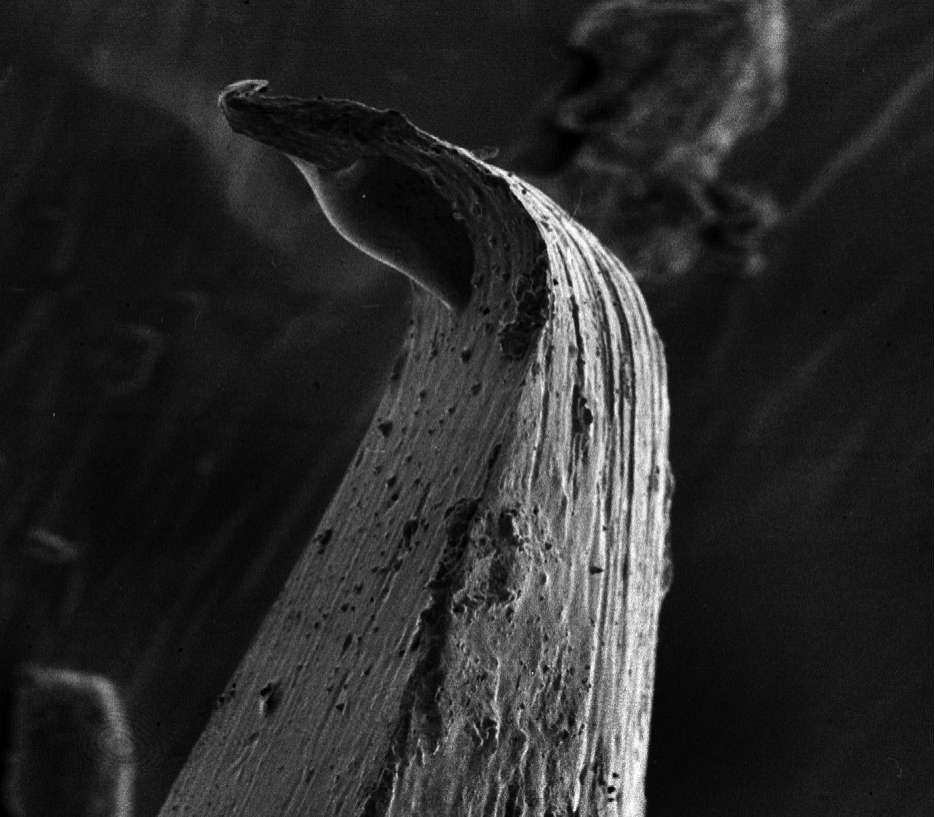
Control sample showing damaged/deformed bevel tip (500x).

Under SEM, 7 of the 93 clinical samples contained excessive soft tissue debris sufficient enough to obscure the extent of needle tip deformation and hence were excluded from the analysis. Additionally, 3 samples of the 10 unused control needle tips showed varying levels of deformation which may be attributed to the manufacturing process (Figs 2 and 3).

All needles examined after having been used for an IANB showed evidence of needle tip deformation.

Bevel orientation during IANB delivery had no significant correlation to the resulting direction of needle tip distortion in terms of inward or outward facing deformation (*P* value = 0.8787, unpaired *t* test, Table 1). Similarly, there was no significance demonstrated between bevel orientation and the angular degree of deformation (*P* value = 0.0752, unpaired *t* test).

**Table 1:** Results of bevel orientation vs direction of deformation

Bevel orientation	Inward deformation	Outward deformation	Needles with debris (excluded)	Total needles
Towards ramus	25	24	2	51
Away from ramus	21	16	5	42
Percentage (excluding debris needles)	53%	47%	‐	100% (of 86)

**Figure 4:**
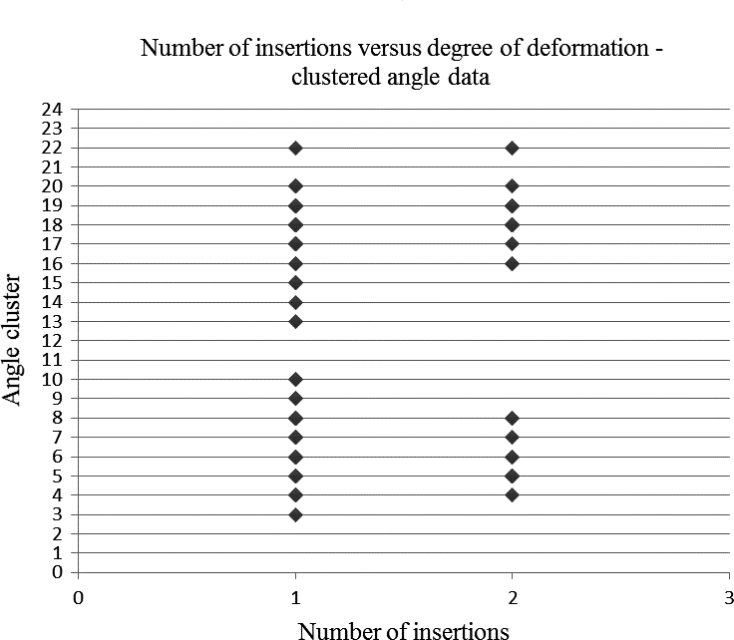
Number of insertions vs degree of deformation – clustered angle data set.

**Figure 5:**
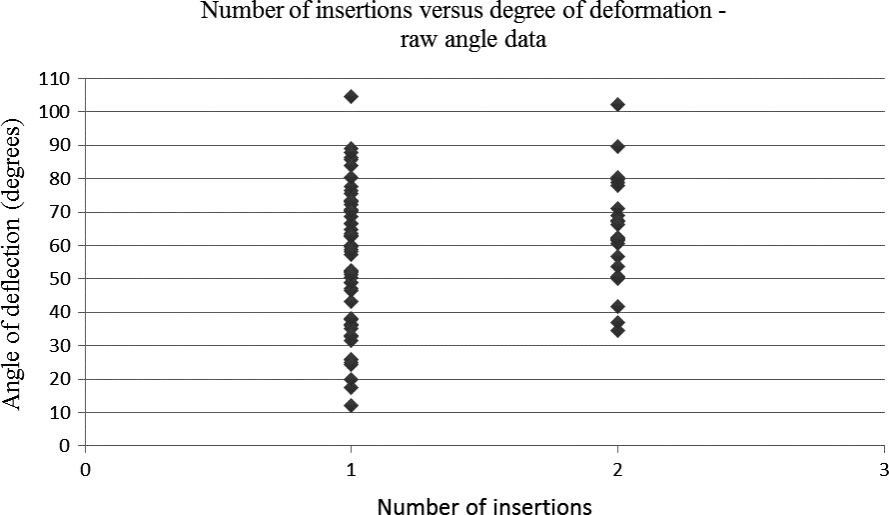
Number of insertions vs degree of deformation – raw angle data set.

The results did, however, demonstrate an association between the number of needle insertions and the degree of needle tip deformation for the clustered angle data set (*P* < 0.0001) (Fig. 4 Table 2) and not for the raw angle data set (*P* = 0.0752) (Fig. 5).

**Table 2:** Angle groups in clustered angle data set

Angle cluster	Angle range in degrees (positive values = deformation towards lumen)
1	−110 to 101
2	−100 to 91
3	−90 to 81
4	−80 to 71
5	−70 to 61
6	−60 to 51
7	−50 to 41
8	−40 to 31
9	−30 to 21
10	−20 to 11
11	−10 to 1
12	0 to 10
13	11 to 20
14	21 to 30
15	31 to 40
16	41 to 50
17	51 to 60
18	61 to 70
19	71 to 80
20	81 to 90
21	91 to 100
22	101 to 110

## Discussion

Previous clinical human studies have consistently confirmed the presence of needle tip deformation following the use for dental local anaesthesia 3, 4, 6. There is a sparsity of information, however, relating to the influence of bevel orientation on this deformation during standard IANB administration where bone is contacted. Furthermore, given the lack of consensus regarding whether it is best to face the needle bevel towards or away from the mandibular ramus, it would be useful to provide information to guide clinical practice in this domain. The study results were unable to demonstrate any significant correlation between bevel orientation and either the direction or the extent of needle tip deformation. This result is not completely surprising however, given the well‐documented complexity and variability of the soft and hard tissue anatomy of the pterygomandibular space 8. Furthermore, many additional potential confounders come into play, which are difficult to control for in such a study.

Firstly, the study is complicated by the influence of manufacturing limitations as evidenced by the SEMs (Figs 2 and 3) showing that of the 10 needle tip controls, 3 showed some evidence of prior deformation. This is not unique to the particular needle type used for this study as it appears elsewhere in the literature where 4 different brands of dental needles exhibited between 4 and 9 degrees of deformation under the SEM 3. Conversely, however, in another dental needle SEM study, no needle tip deformation was recorded amongst 10 controls, although it is unclear whether they were all from a single manufacturer 4. Our study was unable to measure the contributory effect of any pre‐existing needle deformation due to reasons of contamination and sterility.

All needles, following at least one insertion, displayed needle tip deformation after bone contact which seems intuitive and is similar to results in another investigation where 97% of needle tips were also affected 3. It is worth noting, however, that in another study, bevel tip deformation did not increase following bone contact beyond what was attributed to soft tissue caused deformation 7. The aforementioned study used only light microscopy at 40x and 100x power which is considerably less than other SEM studies and this may help explain the inconsistency in results.

Our study results showed no association between bevel orientation relative to the ramus and the direction or the degree of angular deformation. This differs from a previous animal simulation study that suggested that there was a tendency for deformation away from the lumen when the needle bevel was oriented away from the bone 9. This may be partly explained by other research that suggests the presence of considerable variation in mineral bone density from patient to patient 10 as well as in different parts of the mandible itself within the same individual 11. This density differential together with differences in the angular approach and contact angle of the needle tip together with needle elasticity may have affected results.

The raw angle data set (Fig. 5) was unable to demonstrate significance between the number of insertions and the degree of needle tip deformation (*P* = 0.0752). This is likely attributable to the limited size of the data set and angle spread of results. It is for this reason that a clustered angle approach was taken with results grouped into 10‐degree increments, thus yielding a strong significant association (*P *< 0.0001) between the angular degree of deformation and the number of insertions. It can therefore be confirmed by this study that following bone contact during delivery of a standard IANB, needle tip deformation occurs and is made worse with subsequent reinsertion of the same needle. Despite the many potential confounders which might affect the direction and/or severity of needle tip distortion following IANB delivery, it appears prudent practice to remind dentists’ to routinely undertake needle replacement between successive IANBs. This in turn may help maintain a more patent needle tip and likely help reduce potential postoperative complications, previously reported in the literature, such as trismus, paraesthesia, pain and tissue damage 3, 4, 5. Furthermore, there appears to be no benefit in terms of whether it is best to orientate the bevel facing towards or away from the ramus during IANB delivery as this does not seem to substantially alter either the direction or the degree of needle tip deformation.

The study results may have been affected by several confounders which could be considered in future investigations. Firstly, there may be differences in needle tip patency which could vary between different manufacturers and their choice of materials, bevel angle, metal thickness and bore size. Inclusion of additional needle manufacturers may be beneficial. Secondly, study limitations such as sample size, the unequal number of samples from each dentist, patient and technique variability per clinician, sample preparation and SEM measurement errors may have also affected results. There appears no general consensus as to the most appropriate SEM magnification for such studies and at what point a needle tip deformity becomes clinically relevant. It would be beneficial for future investigations in this area to consider including postoperative outcome measures such as injection site pain and trismus, relative to direction and extent of needle tip deformation.

## Conclusion

During the delivery of a standard technique IANB, there appears to be no benefit, in minimising needle tip deformation, by having the bevel face either towards or away from the mandibular ramus. There is strong evidence to suggest that needle tip deformation is made worse following subsequent injections and it may be the best practice to routinely change needles between subsequent IANB injections when contact is made with bone.

## Disclosure

No conflicts of interest or financial interests declared.

## Declaration

All authors contributed significantly to various elements of the study including study design, data collection, analysis and manuscript preparation.
